# The Effect of Insecticide Synergists on the Response of Scabies Mites to Pyrethroid Acaricides

**DOI:** 10.1371/journal.pntd.0000354

**Published:** 2009-01-06

**Authors:** Cielo Pasay, Larry Arlian, Marjorie Morgan, Robin Gunning, Louise Rossiter, Deborah Holt, Shelley Walton, Simone Beckham, James McCarthy

**Affiliations:** 1 Queensland Institute of Medical Research and Australian Centre for International and Tropical Health, University of Queensland, Brisbane, Queensland, Australia; 2 Wright State University, Dayton, Ohio, United States of America; 3 New South Wales Department of Primary Industries, New South Wales, Australia; 4 Menzies School of Health Research, Institute of Advanced Studies, Charles Darwin University, Darwin, Northern Territory, Australia; 5 Monash University, Clayton, Victoria, Australia; Liverpool School of Tropical Medicine, United Kingdom

## Abstract

**Background:**

Permethrin is the active component of topical creams widely used to treat human scabies. Recent evidence has demonstrated that scabies mites are becoming increasingly tolerant to topical permethrin and oral ivermectin. An effective approach to manage pesticide resistance is the addition of synergists to counteract metabolic resistance. Synergists are also useful for laboratory investigation of resistance mechanisms through their ability to inhibit specific metabolic pathways.

**Methodology/Principal Findings:**

To determine the role of metabolic degradation as a mechanism for acaricide resistance in scabies mites, PBO (piperonyl butoxide), DEF (S,S,S-tributyl phosphorotrithioate) and DEM (diethyl maleate) were first tested for synergistic activity with permethrin in a bioassay of mite killing. Then, to investigate the relative role of specific metabolic pathways inhibited by these synergists, enzyme assays were developed to measure esterase, glutathione S-transferase (GST) and cytochrome P450 monooxygenase (cytochrome P450) activity in mite extracts. A statistically significant difference in median survival time of permethrin-resistant *Sarcoptes scabiei* variety *canis* was noted when any of the three synergists were used in combination with permethrin compared to median survival time of mites exposed to permethrin alone (p<0.0001). Incubation of mite homogenates with DEF showed inhibition of esterase activity (37%); inhibition of GST activity (73%) with DEM and inhibition of cytochrome P450 monooxygenase activity (81%) with PBO. A 7-fold increase in esterase activity, a 4-fold increase in GST activity and a 2-fold increase in cytochrome P450 monooxygenase activity were observed in resistant mites compared to sensitive mites.

**Conclusions:**

These findings indicate the potential utility of synergists in reversing resistance to pyrethroid-based acaricides and suggest a significant role of metabolic mechanisms in mediating pyrethroid resistance in scabies mites.

## Author Summary

Synergists are commonly used in combination with pesticides to suppress metabolism-based resistance and increase the efficacy of the agents. They are also useful as tools for laboratory investigation of specific resistance mechanisms based on their ability to inhibit specific metabolic pathways. To determine the role of metabolic degradation as a mechanism for acaricide resistance in human scabies, PBO (piperonyl butoxide), DEF (S,S,S-tributyl phosphorotrithioate) and DEM (diethyl maleate) were used with permethrin as synergists in a bioassay of mite killing. A statistically significant difference in survival time of permethrin-resistant *Sarcoptes scabiei* variety *canis* was noted when any of the three synergists were used in combination with permethrin compared to survival time of mites exposed to permethrin alone (p<0.0001). These results indicate the potential utility of synergists in reversing tolerance to pyrethroid-based acaricides (i.e. the addition of synergists to permethrin-containing topical acaricide cream commonly used to treat scabies). To further verify specific metabolic pathways being inhibited by these synergists, enzyme assays were developed to measure esterase, glutathione S-transferase (GST) and cytochrome P450 monooxygenase activity in scabies mites. Results of *in vitro* enzyme inhibition experiments showed lower levels of esterase activity with DEF; lower levels of GST activity with DEM and lower levels of cytochrome monooxygenase activity with PBO. These findings indicate a metabolic mechanism as mediating pyrethroid resistance in scabies mites.

## Introduction

Scabies is an infectious skin disease caused by the ectoparasite, *Sarcoptes scabiei*. The mite lives in the skin of hosts where it passes through a series of life stages (eggs, larva, protonymph, tritonymph, and adult). A severe manifestation of the disease i.e., crusted scabies can occur which may predispose to streptococcal pyoderma and the subsequent development of acute post streptococcal glomerulonephritis, acute rheumatic fever and rheumatic heart disease [1]. Treatment generally entails the application of topical creams for classical scabies, while oral ivermectin is recommended for crusted scabies [1]. Permethrin, used at a concentration of 5%, is the active component of topical creams commonly used to treat the disease. Since its introduction in Australia in 1994 for the treatment of scabies, it has been widely used in endemic communities in mass treatment programs [2]. Recent evidence from a prospective study of *in vitro* acaricide sensitivity has demonstrated increased tolerance to permethrin of scabies mites collected from indigenous communities across northern Australia (unpublished data).

Pyrethroids constitute one of the most important classes of insecticide, accounting for 17% of the world insecticide market [3]. Their intensive use worldwide over the last 30 years has led to the development of resistance in many arthropods, such that resistance now constitutes a serious threat to many programmes for control of pests and ectoparasites in agriculture, veterinary and human practice.

Various resistance mechanisms such as behavioural (avoidance of treated surfaces), physiological (reduced penetration and/or increased excretion of insecticides), reduced sensitivity of target site (by target alteration) and metabolic degradation (by hyperproduction of enzymes) mediate pesticide resistance of arthropods. Target site insensitivity and metabolic degradation have been demonstrated to play major roles in conferring resistance to pyrethroids in some arthropods. In the German cockroach, *Blattella germanica,* pyrethroid resistance has been demonstrated to be mediated by multiple mechanisms including target site insensitivity, decreased cuticular penetration, and enhanced metabolism by monooxygenases and esterases [4]. The same is true for cattle ticks, a class of arthropods more closely related to scabies mite, where a point mutation in the *kdr* gene and metabolic degradation by esterases have both been identified as mediators of pyrethroid resistance [5]. Recently, pyrethroid resistance in Italian strains of peach potato aphid, *Myzus persicae* has been shown to be conferred by both mutations in the *kdr* gene and hyperproduction of esterases [6].

An effective way to manage pesticide resistance is the coformulation of synergist with the pesticide to counteract metabolic resistance. Synergists act by blocking metabolic pathways that would otherwise break down pesticides, thus restoring susceptibility to the agent. Piperonyl Butoxide (PBO) is co-formulated with insecticides such as carbaryl, methomyl, fenvalerate, permethrin, parathion, malathion and dimethoate; S,S,S-tributyl phosphorotrithioate (DEF) is co-formulated with malathion and permethrin; and Diethyl maleate (DEM) is co-formulated with parathion, malathion and dimethoate [7]. For example, PBO has been used as a synergist to pyrethrins in commercially available treatment for head lice (caused by *Pediculus humanus capitis*) to counteract resistance. Examples of over-the –counter treatment products for headlice are: Pronto (33% pyrethrins and 4% PBO) (Del Laboratories, Uniondale, NY), RID (33% pyrethrins and 4% PBO) (Bayer, Morristown, NJ), A-200 (33% pyrethrins and 4% PBO) (Hogil Pharmaceutical Corp., Purchase, NY) available in the US and BanLice Mousse (pyrethrin 1.65 mg and 16.5 mg PBO) (Pfizer) available in Australia [8].

In addition, insecticide synergists have been used to investigate resistance mechanisms and to identify the specific metabolic pathways targeted. Tolerance of honey bees, *Apis mellifera*, to pyrethroids is largely reversed by PBO and at low levels by DEF, suggesting a significant role of cytochrome P450s and a lesser role of esterases in detoxification of the chemical [9]. However, the metabolic routes blocked by synergists are not yet fully understood and maybe dependent on the species of arthropods. For example, although initial evidence in the 1970's suggested that PBO acts as a specific inhibitor of monooxygenases (P450s), recently, esterases of pyrethroid resistant strains of the cotton bollworm, (*Helicoverpa armigera*) were shown to be inhibited by PBO [10]. In a study conducted with insecticide resistant strain of the German cockroach, *Blatella germanica*, PBO and DEF were tested as synergists to propoxur (a carbamate insecticide). Both were shown to inhibit cytochrome P450 monooxygenases at different levels [11], thus, suggesting that this enzyme family is primarily responsible for the metabolism of the pesticide.

Previously, we identified a single nucleotide polymorphism (SNP) in the *kdr* gene associated with permethrin resistance in a population of scabies mites [12]. In this study, the same strain of scabies mites, *Sarcoptes scabiei* var *canis* was used to investigate the role of metabolic degradation as a contributory mechanism to observed permethrin resistance. PBO, DEF and DEM were tested as synergists to permethrin to determine if susceptibility to the acaricide can be restored, and to explore their activity in inhibiting specific metabolic pathways.

## Materials and Methods

### Source of Mites

Live scabies mites (*Sarcoptes scabiei*) were collected from two sources: permethrin naïve mites (*Sarcoptes scabiei* var. *suis*) were collected from a colony maintained on pigs (Kelly, A. unpublished) while permethrin resistant mites (*Sarcoptes scabiei* var. *canis*) that had been maintained under permethrin treatment for many years were collected from rabbits [12],[13]. The establishment of mite colony on pigs was approved by the Animal Ethics Committee of the Department of Primary Industries and Fisheries, Queensland adhering to the Institution's guidelines for animal research. Rabbits were maintained under a protocol approved by the Wright State University Laboratory Animal Care and Use Committee in adherence to institutional guidelines for animal husbandry. Mites were used live immediately in bioassays or collected and stored at −80°C until use in enzyme inhibition assays.

### Chemicals and Reagents

Synergists used in the bioassays and biochemical assays were: Piperonyl Butoxide (PBO- Sigma, Milwaukee, WI); Diethyl Maleate (DEM- Sigma, Milwaukee, WI); and S,S,S tributylphosphorotrithioate (DEF- ChemServices West Chester, PA). All other chemicals and reagents used in enzyme assays were purchased from Sigma.

Reagents used in the bioassays were: 5% Permethrin (Elimite Cream- DSM Pharmaceuticals Inc, Greenville, NC); Benzyl Benzoate (Ascabiol Lotion- Aventis Pharma Pty Ltd, Lane Cove, NSW); and mineral oil (Sigma).

### Bioassays

In preliminary experiments, a small number of mites (30/synergist) were exposed to each synergist alone (serially diluted starting from 300 mM) to determine maximum concentration that the mites could tolerate and remain alive for 24 hours (data not shown). The result of this preliminary experiment led us to select a concentration (30 mM) of each synergist for use in subsequent bioassays.

Acaricide bioassays were performed on live scabies mites as previously described [12] with some modifications. Briefly, acaricide and synergists were spread thinly on plastic petri dishes and then groups of female scabies mites were placed in the dish. Mites were exposed to 5% Permethrin alone, 30 mM of each synergist alone (PBO, DEF and DEM), 30 mM of each synergist alone (PBO, DEF or DEM) plus 5% Permethrin, Benzyl Benzoate (positive control acaricide) and mineral oil (negative control) for 24 hours. Petri dishes containing the mites were held in a 28°C incubator at 100% relative humidity. The mites were held at 28°C because lower temperature and higher relative humidity have been found to favour survival of mites outside the host [14]. Individual mites were observed at hourly intervals for 24 hours under a microscope. The time of death, defined as complete absence of movement and a cessation of peristalsis of the gut was recorded. A total of 100 mites in each treatment group were assayed in each set of synergist bioassay. Survival times of mites in acaricide and controls were analysed using Survival Analysis in Graph Pad Prism 4. Kaplan-Meier survival curves of mites in each treatment group were generated and statistical significance of differences in survival curves determined by logrank test.

### Enzyme Inhibition Assays

#### Preparation of mite homogenate

Scabies mites were homogenized on ice in 100 ul 0.02M phosphate buffer pH 7.0 (esterase assay), 0.05M Tris-HCl buffer pH 7.5 (GST assay) or 0.1M sodium phosphate buffer pH 7.6 (cytochrome P450 assay), using a hand-held battery operated homogenizer (Kontes Glass Company, Vineland, NJ). After homogenization, the tubes were spun at 13,000×g at 4°C for 5 minutes. Mites used for cytochrome P450 assay were spun at 1000×g at 4°C for 15 mins. The protein concentration of the lysate was determined using the Nanodrop ND-100 Spectrophotometer (Nanodrop Technologies, Wilmington, DE, USA). Mite homogenates used in all enzyme assays were normalized to 2 mg/ml protein concentration.

#### *In vitro* enzyme inhibition

Twenty microliters of mite homogenate supernatant was incubated with varying dilutions (30 mM, 15 mM, 8 mM and 4 mM) of enzyme inhibitors (PBO, DEF and DEM) for 30 minutes at room temperature. Enzyme assays were performed as outlined below:

### Esterase Assay

Esterase activity was determined using alpha-napthyl acetate as the substrate which forms a diazo-dye complex with Fast Blue RR Salt. Colorimetric formation of the complex is directly proportional to esterase activity and is measured continuously in a kinetic assay as described by Gunning et al, 1996 [15] with some modifications:

To mite homogenate (with and without inhibitor) set up in triplicate, 200 ul of substrate (6 mg of Fast Blue dissolved in 10 ml of 0.2M phosphate buffer pH 6.0+100 ul of 100 mM alpha-naphthyl acetate in acetone) was added. Change in absorbance was measured at 450 nm at 14 second intervals for 10 minutes in a kinetic microplate reader (POLARstar Optima, BMG LabTech). As an assay control, esterase from rabbit liver, diluted 1∶10 ((0.10 mg/ml) and 1∶100 (0.01 mg/ml) was included in each experiment.

### Glutathione S-Transferase Assay

GST activity was measured by using monochlorobimane (MCB) as the substrate which forms a stable fluorescent conjugate with reduced glutathione. The enzymatic conversion of MCB to bimane-glutathione is measured in an endpoint fluorescence assay as described by Nauen and Stumpf, 2002 [16] with some modifications:

To mite homogenate (with and without inhibitor) set up in triplicate, 200 ul of substrate (3 mg reduced glutathione dissolved in 10 ml of 0.05M Tris-HCl buffer pH 7.5+500 ul of 1.5 mg/ml of monochlorobimane (MCB) in methanol) was added. Mite homogenate and substrate were incubated for 20 minutes at room temperature. Fluorescence intensity was read on a microplate reader (Polarstar) with 465 Emission and 390 Excitation filters. As an assay control, GST from equine liver, diluted 1∶10 (0.10 mg/ml) and 1∶100 (0.01 mg/ml) was included in each experiment.

### Cytochrome P450 Assay

Cytochrome P450 activity was measured by using 7-ethoxy coumarin as the substrate which results in a fluorescent 7-hydroxycoumarin. The O-deethylation of 7-EC is measured in an endpoint fluorescent assay as described by Stumpf and Nauen, 2001 [17] with some modifications:

To mite homogenate (with and without inhibitor) set up in triplicate, 40 ul of 0.1M sodium phosphate buffer pH 7.6, 2 ul of 20 mM 7-EC in acetone and 10 ul of 10 mM Aqueous NADPH was added. The enzyme and substrate reaction mixture was incubated for 30 mins at 30°C with shaking. Excess NADPH was removed by adding 10 ul of 100 mM oxidized glutathione and 13 ul of 10 U/ul of glutathione reductase and the reaction incubated for 10 mins at room temperature. Fluorescence intensity was read immediately after incubation using 465 Emission and 390 Excitation filters in a microplate reader (Polarstar). As an assay control, human Cytochrome P450 isoform diluted 1∶10 (0.10 mg/ml) and 1∶100 (0.01 mg/ml) was included in each experiment.

## Results

### Bioassays

**Figure 1:**
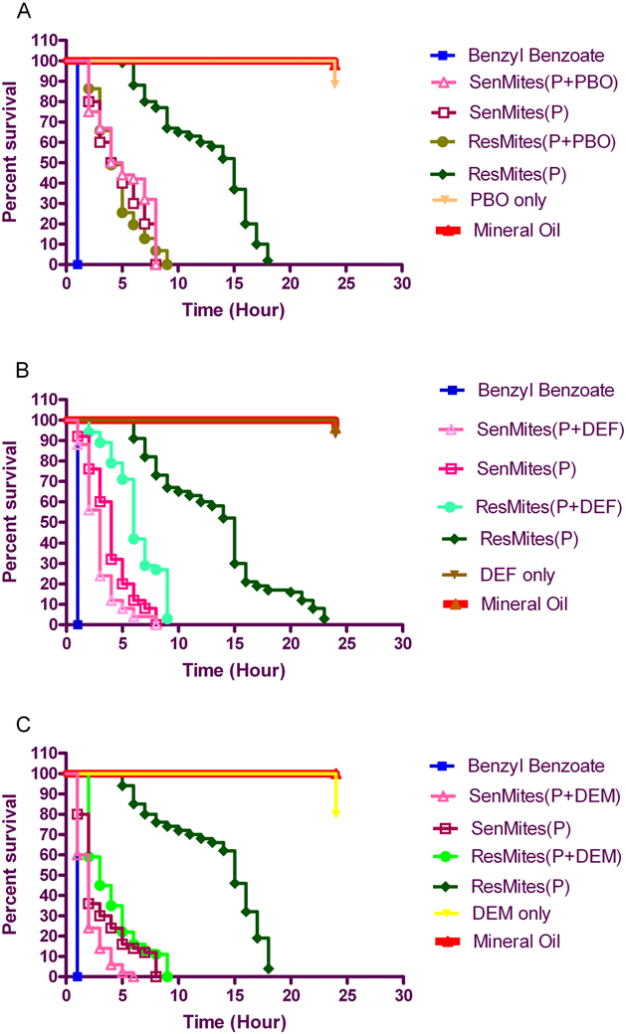
Survival of Permethrin (P) Resistant (Res) and Sensitive (Sen) Mites with or without Synergists. A. Survival of scabies mites using PBO (Piperonyl Butoxide) as synergist to permethrin. B. Survival of scabies mites using DEF (S,S,S-tributylphosphorotrithioate) as synergist to permethrin. C. Survival of scabies mites using DEM (Diethyl Maleate) as synergist to permethrin. Benzyl Benzoate was used as positive control and Mineral Oil was used as negative control. Synergist (PBO, DEF and DEM) only controls were also included in the bioassays.

Survival time of both permethrin susceptible and permethrin resistant mites over the 24-hour exposure to acaricide and acaricide+/−synergist are shown in Figure 1A–C and median survival times are listed in Table 1. Both resistant and sensitive mites (100%) died within minutes of contact with benzyl benzoate (positive control acaricide) while 98% of mites exposed to mineral oil (negative control) and 97% of mites exposed to synergists alone remained alive throughout the 24-hour observation period. A significant reduction in survival time of resistant mites was observed (p<0.0001) when exposed to permethrin in combination with PBO (median survival = 4 hours) as compared to survival time when exposed to permethrin alone (median survival = 15 hours) (Fig. 1A). A similar result was observed when resistant mites were exposed to permethrin in combination with DEF (Fig. 1B), with a median survival reduced from 15 to 6 hours (p<0.0001). Similarly, the median survival of the resistant mites was significantly reduced to 3 hours (p<0.0001) when DEM was used as a synergist (Fig. 1C). In contrast, median survival time of permethrin susceptible pig scabies mites exposed to permethrin and permethrin in combination with PBO, DEF and DEM did not show significant differences (p = 0.128; p = 0.465; p = 0.220, respectively). Of note, the median survival (4 hours) of resistant mites in permethrin+PBO was identical to the median survival of sensitive mites exposed to permethrin alone suggesting complete reversal of the resistance phenotype using PBO as synergist.

**Table 1:** Median survival (hrs) of sensitive and resistant mites in permethrin with and without synergist.

Treatment	Sensitive mites	Resistant mites
Permethrin	4	15
P+PBO	4	4*
P+DEF	3.5	6*
P+DEM	2	3*

*: Significant differences in median survival of resistant mites in permethrin and permethrin+synergist by log rank test (*p*<0.0001).

### Enzyme Inhibition Assays

To investigate possible metabolic pathways inhibited by synergists, enzyme inhibition assays were performed. Homogenate supernatant from resistant mites were incubated with the synergists (PBO, DEF, DEM) acting as inhibitor and enzyme levels were compared with and without inhibitors. Homogenate supernatants from susceptible mites were also incubated with the synergists for comparison. Enzyme inhibition experiments were repeated several times and showed consistent results. Significant differences in enzyme activities with and without inhibitors were determined by *t* test.

### Esterase Inhibition by Synergists

**Figure 2:**
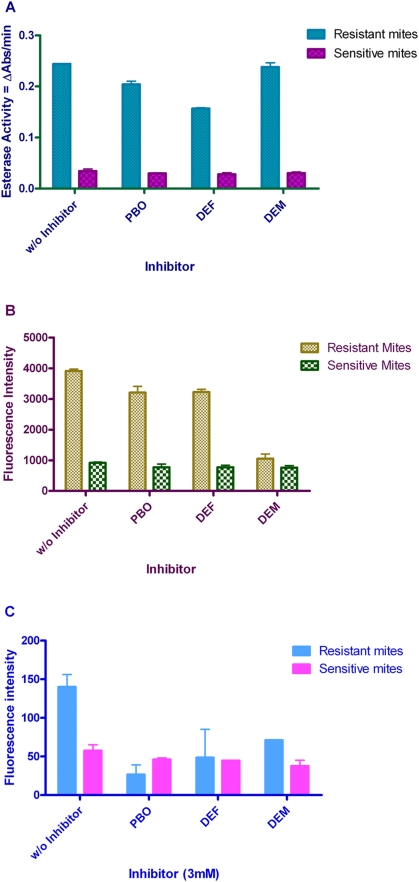
Results of *in-vitro* enzyme inhibition by synergists/inhibitors (30 mM). A. Esterase activity in resistant and sensitive scabies mites in the presence or absence of inhibitor (PBO, DEF and DEM). B. GST activity in resistant and sensitive mites in the presence or absence of inhibitor (PBO, DEF and DEM). C. Cytochrome P450 activity in the presence or absence of inhibitor (PBO, DEF and DEM). *Error bars represent SEM.

**Figure 3:**
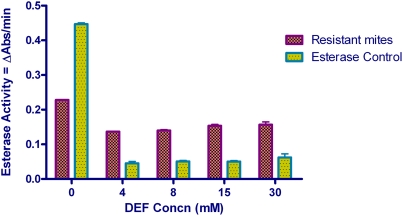
Esterase levels of resistant mites with varying concentrations of DEF. *Error bars represent SEM. **Commercial esterase from rabbit liver was used as assay control.

Esterase activity in resistant scabies mites was significantly inhibited by DEF (36%; p<0.0001), and to lesser degree by PBO (16%; p = 0.006). Inhibition of esterase activity in resistant mites by DEM was unremarkable (0.6%; p = 0.507). A decrease in esterase activity was likewise observed in susceptible mites with PBO, DEF and DEM. However, the decrease was not statistically significant (p = 0.337; p = 0.280; p = 0.443, respectively) (Fig. 2A). Varying concentrations of DEF did not alter esterase activity in resistant mites (Fig. 3). Esterase activity levels in resistant mites was 7-fold (p<0.0001) higher than in sensitive mites.

### GST Inhibition by Synergists

**Figure 4:**
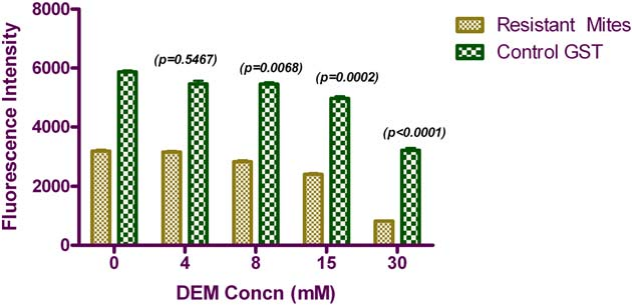
GST levels of resistant mites with varying concentrations of DEM. *Error bars represent SEM. **Commercial GST from equine liver was used as assay control.

GST levels of resistant scabies mites were significantly inhibited by DEM (73%; p<0.0001) and to lesser degree by PBO (18.0%; p = 0.017) and DEF (17.5%; p = 0.018). PBO, DEF and DEM decreased GST activity in sensitive mites but the decrease was not statistically significant (p = 0.219; p = 0.094; p = 0.065, respectively) (Fig. 2B). A dose-dependent inhibition of GST activity by DEM was also observed in the resistant mites with maximum effect at 30 mM concentration. GST activity was observed to return to uninhibited level at 4 mM concentration of DEM (Fig. 4). A 4-fold (p<0.0001) increase in GST levels was observed in resistant mites compared to sensitive mites.

### Cytochrome P450

Cytochrome P450 monooxygenase activity in resistant mites was significantly inhibited by PBO (81%; p<0.0001), DEF (65%; p = 0.0073) and DEM (49%; p = 0.0003) at 3mM concentration. In sensitive mites, CYP450 monooxygenase activity is significantly inhibited by DEM (34%; p<0.02); by DEF (24%; p = 0.03) and PBO (21%; p = 0.04) at the same concentration of inhibitor (Fig. 2C). Results of CYP450 inhibition in resistant and sensitive mites are unremarkable at 30 mM concentration of inhibitor. A 2.4-fold (p = 0.0002) increase in cytochrome P450 monoxygenase activity was observed in resistant mites compared to sensitive mites.

## Discussion

Detoxifying enzymes and target alteration are equally important mechanisms of insecticide degradation in ticks and mites. While a point mutation in the Vssc gene has been associated with pyrethroid resistance in two strains of cattle ticks, *Boophilus microplus*, high levels of esterase activity have been observed in another two strains in the absence of this mutation [5]. Resistance to tau-fluvalinate in the honeybee mite, *Varroa destructor* has been associated with sodium channel insensitivity as well as elevated cytochrome P450s and high levels of esterases [18],[19].

We have previously addressed target alteration as a mechanism of pyrethroid resistance in scabies mites. A non-synonymous SNP was identified in the *kdr* gene associated with permethrin resistance, in the population of *Sarcoptes scabiei* var *canis* mites studied here and developed a highly sensitive high resolution melt (HRM) assay for its detection [12]. In this study, further investigation was done to verify if the resistance phenotype may also be mediated by detoxifying enzymes, hypothesizing that multiple mechanisms may be present as had been reported in the cattle ticks and honeybee mites.

To better understand metabolic mechanisms of pyrethroid resistance in *Sarcoptes scabiei* mites, commonly used synergists PBO, DEF and DEM were used in acaricide bioassays. Enzyme assays were performed using three biochemical markers such as esterases, GSTs and cytochrome P450s linked to insecticide resistance to further define specific metabolic pathways inhibited in a population of resistant scabies mites.

In this study, significantly higher levels of esterase, GST and cytochrome P450 monooxygenase activity in resistant mites were observed when compared to sensitive mites suggesting metabolic degradation as a mechanism of pyrethroid resistance in this population of scabies mites. It was also observed that PBO, DEF and DEM when used as synergists to permethrin in bioassays, significantly decreased the survival times of resistant mites. These findings are in agreement with results of enzyme inhibition experiments that showed inhibition of cytochrome P450 monooxygenase activity (81%) by PBO, inhibition of GST activity (73%) by DEM and inhibition of . esterase activity (36%) by DEF when homogenate supernatant of resistant mites were incubated with the synergists. These results suggest possible roles of GSTs, esterases and cytochrome P450 monooxygenases in detoxifying permethrin in this population of resistant mites.

In initial assays of cytochrome P450 we failed to detect enzyme activity in both sensitive and resistant mites using homogenates spun at high speed. However, in subsequent assays using mite homogenate spun at a much lower speed (1000×g), positive results were observed [20]. This change in methodology is based on the hypothesis that mites maybe similar to insects in that monooxygenase activity is primarily present in the microsomes; released out of the endoplasmic reticulum by mechanical homogenisation [21] and lower centrifugation allows the microsomes to stay in solution to be used in the assay. The cytochrome levels detected in resistant mites was significantly inhibited by all three synergists with the greatest inhibition achieved by PBO at a much lower concentration. These findings suggest oxidative metabolism catalysed by P450's may also be an important route for the detoxification of pyrethroids in this population of resistant mites.

Overall, these findings have demonstrated potential utility of synergists in reversing resistance to acaricide and validated metabolic mechanisms of pyrethroid resistance in scabies mites. The results of this study also support our hypothesis that permethrin resistance in this population of scabies mites may be mediated by multiple mechanisms including target alteration, as demonstrated in our previous study [12], and by metabolic degradation, as demonstrated in this study.

The addition of a synergist to topical creams containing permethrin as the active ingredient may result in better clinical management of acaricide resistant scabies. Improved management of permethrin resistant head lice infestations has already been achieved by adding PBO as a synergist to pyrethrin based head lice shampoos and are now available as over the counter products [8].

This work now leads us to consider undertaking in-vitro susceptibility and pesticide synergist studies on clinical isolates from scabies-endemic communities in remote Aboriginal communities in Northern Australia where mass drug administration is underway. These studies will determine the potential role of metabolic degradation as a mechanism of permethrin resistance in human scabies mites.
